# Extending Situated Language Comprehension (Accounts) with Speaker and Comprehender Characteristics: Toward Socially Situated Interpretation

**DOI:** 10.3389/fpsyg.2017.02267

**Published:** 2018-01-24

**Authors:** Katja Münster, Pia Knoeferle

**Affiliations:** Department of German Studies and Linguistics, Humboldt University of Berlin, Berlin, Germany

**Keywords:** real-time language processing, Coordinated Interplay Account, social context, comprehender, speaker, psycholinguistics, sociolinguistics

## Abstract

More and more findings suggest a tight temporal coupling between (non-linguistic) socially interpreted context and language processing. Still, real-time language processing accounts remain largely elusive with respect to the influence of biological (e.g., age) and experiential (e.g., world and moral knowledge) comprehender characteristics and the influence of the ‘socially interpreted’ context, as for instance provided by the speaker. This context could include actions, facial expressions, a speaker’s voice or gaze, and gestures among others. We review findings from social psychology, sociolinguistics and psycholinguistics to highlight the relevance of (the interplay between) the socially interpreted context and comprehender characteristics for language processing. The review informs the extension of an extant real-time processing account (already featuring a coordinated interplay between language comprehension and the non-linguistic visual context) with a variable (‘ProCom’) that captures characteristics of the language user and with a first approximation of the comprehender’s speaker representation. Extending the CIA to the sCIA (social Coordinated Interplay Account) is the first step toward a real-time language *comprehension* account which might eventually accommodate the socially situated communicative interplay between comprehenders and speakers.

## Introduction

The way we interact socially with others is not only influenced by explicit culturally and socially agreed-upon constructs, but seems to also implicate implicit behavioral mechanisms. For instance, synchronous actions and shared goals implicitly strengthen the feeling of group membership and improve cooperation among its members (Wiltermuth and Heath, 2009). Imitating an interlocutor’s verbal behavior can also improve that interlocutor’s behavior toward us. When a waiter repeated the order of a customer, tip size increased compared to when he didn’t repeat the order (Baaren et al., 2003). Underlying this effect is a link between perception and action which “[…] exists, at least in part, as a kind of natural ‘social glue’ that produces emphatic understanding and even greater liking between people, without their having to intend or try to have this happen” (Chartrand and Bargh, 1999, p. 897). This link extends to visual perception: Even the mere knowledge of shared perception, i.e., knowing that another person is looking at the same images, changes the way we look at them (Richardson et al., 2012). The verbal and non-verbal (re)actions as part of the perception-action link seem to be entwined with social conventions such as ordering food in a restaurant and tipping and arguably serve as a communicative cue to the social world. Moreover, at the level of visual attention (e.g., in an interpersonal version of the Posner spatial cueing paradigm) Gobel et al. (2017) have claimed that people’s visual attention is guided by a mental representation of the social relevance of the environment (but see Staudte et al., 2014). Taking this insight further into language processing, one might postulate that social behavior interacts with (all sorts of real-time) comprehension (and production) processes, and that we must include these interactions in our theories and (ultimately computational) models of language processing (see Zwaan, 2009 for related thoughts on specifying process models of mental simulation in language comprehension and social cognition).

In assessing our postulate, we consider “the processes of encoding and decoding as they relate states of messages to states of communicators” (Osgood and Sebeok, 1954, p. 4), including the mental and brain mechanisms underlying language comprehension and production (Traxler, 2012). Psycholinguistics has examined the link between (language and visual) perception and action (Tanenhaus et al., 1995, see also Tanenhaus, 2007 for a review) and considers language processing in relation to spatial and visual attention, human judgment, and the organization of control and memory systems (Garrett, 2009). By comparison, extant (theoretical and computational) modeling approaches have not yet accommodated effects of the socially interpreted context in (real-time) language processing. Among these models are constraint-based models of language processing (cf., Spivey-Knowlton et al., 1993; MacDonald et al., 1994; McRae et al., 1998), situation models and frameworks (cf., Zwaan and Radvansky, 1998; Zwaan, 2003), embodied and situated theories of cognition (cf., Barsalou, 1999; Myachykov et al., 2014), and processing accounts of situated language comprehension [Coordinated Interplay Account (CIA), Knoeferle and Crocker, 2006, 2007; Knoeferle et al., 2014]. Approaches to dialog interaction, such as Pickering and Garrod’s (2004) ‘interactive alignment’ framework capture the alignment of interlocutors at linguistic levels (e.g., phonological, syntactic, and semantic). But they also consider alignment of situation models (e.g., referential relations), leading to mutual understanding (Pickering and Garrod, 2004), and have included social perspectives (Garrod and Pickering, 2009; Pickering and Garrod, 2009; see Kuhlen and Abdel Rahman, 2017 on how having a partner affects lexical retrieval; see Schindler and Kissler, 2017 on social feedback).

Given the insights from social psychology that perception and social behaviors are closely linked, forming “a kind of natural ‘social glue”’ (Chartrand and Bargh, 1999, p. 897) one might – at first blush – be surprised that psycholinguistic theories and models of real-time language processing have paid little attention to social aspects of communication. But upon closer inspection it is perhaps not that surprising. Much psycholinguistic research focuses on compositional syntactic and semantic processes, and the extent to which socially interpreted cues impact these is somewhat unclear. Returning to the tipping-example, maybe increased tipping emerges when a waiter fully repeats a customer’s utterance (but not when he mimics merely the customer’s sentence structure). However, even subtle aspects of language style can imperceptibly influence communication and social outcomes such as relationship success (Niederhoffer and Pennebaker, 2002), lending some credence to postulating a link between social behavior and core comprehension processes also. Recent empirical evidence, moreover, suggests that a socially interpreted context (as e.g., provided by the speaker’s facial expression or voice) can modulate not just relationship success or tipping behavior but even real-time comprehension processes (i.e., Van Berkum et al., 2008; Carminati and Knoeferle, 2013, 2016).

The present article focuses on extending extant approaches to real-time situated language comprehension with effects of the socially interpreted context by explicitly integrating comprehender characteristics and representations of the speaker (of which more below, see (1) to (3)). Note that we use the term ‘comprehender’ instead of ‘listener’ to capture effects of the language user’s characteristics on both spoken and written language comprehension (see Guerra and Knoeferle, 2014, 2017; Knoeferle et al., 2014 on how to accommodate visual context effects in written language comprehension; see Knoeferle and Crocker, 2006, 2007 for accommodating visual context effects in spoken language comprehension). In integrating effects of the socially interpreted context on real-time language processing into a theoretical account, we must make several decisions. First, we could focus on either society as a group, subgroups, or on individuals. Labov (1972) and many other sociolinguists (see e.g., Gumperz, 1971; Chambers, 2002; Wardhaugh, 2006) focus on society as a group, and on language variation due to social and cultural shifts. Indeed, society has been characterized as “[…] any group of people who are drawn together for a certain purpose or purposes” (Wardhaugh, 2006, p. 1). Social psychology, by contrast, focuses on (feelings, beliefs, and behaviors of) the individual. Many social psychologists emphasize this sort of ‘microstructure’; yet, individual beliefs, feelings and attitudes are also shaped by the social world individuals live in (the ‘macro structure’). Bar-Tal (2006) argues for uniting these two perspectives to enable a more holistic understanding of social psychology. With regard to language processing, Labov (1972) and Průcha (1972) relatedly argue that studying language use must involve factors such as the IQ, emotional state, verbal memory, age, sex, education, status and ethnicity of the individual. Průcha moreover assumes that the speaker’s attitude, social situation, and intended meaning will all shape his message (for an interactive account focusing on the alignment between interaction partners see Pickering and Garrod, 2004, 2009, 2013; Garrod and Pickering, 2009)^1^.

In the present paper, we focus on the characteristics of subgroups of individuals (since most studies in psycholinguistics report average measures across participants). We ask how the (socially relevant) characteristics (1) and (2) can modulate language comprehension in relation to context (3). Note that we are aware of the work on accent perception and perceptions of non-nativeness (see e.g., Giles and Billings, 2004 for a review). Ultimately, we may extend our approach to also capture non-native language processing; however, this involves decisions about how to model mental representations in the face of bi- or multilingualism that are beyond the remit of this paper.

The (socially relevant) comprehender characteristics capture:

(1) Largely biological comprehender characteristics such as age (and their impact on cognition and behavior); for instance, aging may cause differences in language comprehension.
(2) A comprehender’s experiences over time, leading to a certain education level (e.g., literacy, Mishra et al., 2012), mood (e.g., Van Berkum et al., 2013), stereotypes about groups of people (Rodríguez et al., 2016) or beliefs and world knowledge (e.g., Van Berkum et al., 2009).

The characteristics (1) and (2) can come into play in a specific (linguistic and non-linguistic) context [(3), encompassing for example referents, actions, events, gestures, speaker gaze, emotional facial expression and speaker voice among others, see Knoeferle and Guerra, 2012 on what’s in a visual context; see e.g., Meibauer, 2012 on linguistic context].

The outcome of the comprehender’s language interpretation in context [including effects of (1)–(3)] is **the socially (contextually) situated interpretation**. The extent to which the resulting interpretation is informed by the language user’s characteristics may vary. For instance, considering the non-linguistic visual context, a comprehender may adopt a positive interpretation of a sentence because he has just seen the speaker smile. This effect could be mediated by visual perception (a perceived smile elicits representations such as ‘happy’) or mimicry (the activation of facial muscles involved in smiling links to a representation of ‘happy’), guiding the comprehender toward matching positive sentence valence without activating further social experiences. Alternatively, or in addition, seeing a speaker smile reactivates associated social experiences, which then in turn guide the comprehender toward a positively valenced sentence interpretation, a guidance that may further vary by comprehender age (e.g., Carminati and Knoeferle, 2013, 2016). Considering experiences associated with voice, the comprehender’s knowledge about what is stereotypical for young children (2) activated by the voice of a speaker (3) (e.g., Van Berkum et al., 2008) may also cause him to develop specific expectations regarding the interpretation (and about what will be mentioned next).

In summary, we draw on insights from sociolinguistics, social psychology, and psycholinguistics to enrich an existing account of the interplay between information from the non-linguistic (visual) context and language comprehension. We outline an extension of the Coordinated Interplay Account (CIA, Knoeferle and Crocker, 2006, 2007; Knoeferle et al., 2014) to include (1)–(3) (‘social Coordinated Interplay Account, sCIA’). We motivate the extension of the account via a review of developmental research, extant accounts, models and theories of language comprehension, and evidence from picture choice, reaction time, eye tracking and event-related brain potential (ERP) research.

## Speech, Joint Attention, and Faces: Social Information in Early Child Language

The idea that language processing and the social context are closely linked receives support from research concerning talker identity and speech recognition (Creel and Bregman, 2011). Social conventions even seem to affect vocal tract properties in early infancy. Before children reach puberty, females have higher formant frequencies, i.e., they speak with a higher pitch than males, even though boys and girls do not yet differ physically in vocal tract properties and hence in their fundamental f0 frequency (Perry et al., 2001). These higher formant frequencies for girls may be the consequence of learned gender-typical ‘dialects’ that generate speech pattern differences in males and females even before puberty affects vocal tract properties (Johnson, 2006).

Further support for (familiar) acoustic speech patterns guiding visual attention and behavior comes from infant studies. Neonates, for instance, react differently to the voice of a stranger than to their own mother’s voice (DeCasper and Fifer, 1980). Infants in a preferential looking study additionally preferred to inspect faces which were previously paired with their native language (vs. a foreign language, Kinzler et al., 2007). Moreover, 5-year-olds prefer to be friends with children who speak their native language without an accent (vs. native but accented language vs. foreign language, Kinzler et al., 2009). This suggests that familiarity of speech has implications for social interaction (see also Narayan et al., 2017 for recent evidence on talker discrimination in adults).

Further information in the context can inform language learning. For example, infants as young as 8 months of age learned faster from a facial than an attentional cue (square) in an audio-visual learning task (Yurovsky et al., 2011). Moreover, Yu and Ballard (2007) argue for an integration of information that signals joint attention, such as gaze direction, gesture and facial expressions into statistical learning models. In an analysis of CHILDES video clips of mother–infant interactions, a model including both child-directed prosody and joint attention based on deictic reference outperformed the results of a statistical learning method which only computed word-meaning associations (see also Weisleder and Fernald, 2014 regarding the effects of social interaction on language development). Furthermore, a context permitting joint attention between a child and her caregiver matters for language learning (Kuhl et al., 2003; Kuhl, 2007, see also Tomasello et al., 2005), and joint attention episodes seem positively related to young infants’ vocabulary size (e.g., Tomasello and Todd, 1983; Tomasello and Farrar, 1986). In summary, experience such as voice familiarity and its association with context but also joint attention in social interaction seems to influence (language) learning from a very early age.

## Extant Approaches to Social Effects in Language Processing

How do theories of communication deal with socially elicited behavioral responses? The Communication Adaptation Theory (CAT) by Giles et al. (1991) focuses on the adaptation of interlocutors, e.g., through speech, vocal patterns, gestures, and accents, but also through social norms and social situations. According to CAT, people converge and diverge with their communicative partners. The more the partners’ behavior and speech patterns resemble each other, the more the partners converge and sympathize. Likewise, the linguistic style matching (LSM) that Niederhoffer and Pennebaker (2002) propose, argues that a speaker’s words unconsciously prime a listener’s response, and thus use of words. This resembles the non-linguistic coordination mentioned above and implies, according to Niederhoffer and Pennebaker (2002, p. 339), that conversation partners with matching linguistic styles are similar “[…] in the ways they organize their psychological world.” CAT and LMS both focus on communication as a social phenomenon (governed by an accommodation mechanism). By contrast, the effects of socially interpreted context (3) and comprehender characteristics (1) and (2) have received less attention in models of *real-time* language comprehension.

A connection to social factors can be established in psycholinguistic dialog frameworks: While Pickering and Garrod’s (2004) ‘interactive alignment’ framework focuses on alignment at linguistic levels (e.g., phonological, syntactic, and semantic), it need not be limited to that (Pickering and Garrod, 2004, p. 188) and is open to social perspectives on cognition (Garrod and Pickering, 2009; Pickering and Garrod, 2009). Consider one finding – viz. that infant phonetic learning occurs during active tutor interaction but not during passive television listening (see Kuhl, 2007, 2011). In the alignment framework, listener/learner representations could be more and more aligned with speaker/tutor representations in interactive settings (perhaps interactive tutoring activates comprehension and production mechanisms to a greater extent, strengthening the to-be-learned representations, see Pickering and Garrod, 2013 on the role of forward modeling of production in comprehension, providing a link from comprehension to production).

The indexical hypothesis by Glenberg and Robertson (1999) states that words are linked to the visual context (e.g., objects) and that experience-based object-related knowledge becomes available via that link. However, the authors make no claims regarding the precise integration of this kind of information during language processing. Situation models and frameworks (Zwaan and Radvansky, 1998; Zwaan, 2003), as well as situated theories of cognition (e.g., Myachykov et al., 2014) take world knowledge, the situational context and embodied representations into account with a focus on the nature of representations (embodied vs. abstract, see also Barsalou, 1999 for comprehension processes). These approaches provide valuable insights into the nature of representations but remain underspecified as to their effects on real-time language comprehension (see Zwaan, 2009 on mental simulation in language comprehension and social cognition).

Vice versa, most architecturally restricted, serial language comprehension accounts (e.g., Frazier and Fodor, 1979; Friederici, 2002) include impoverished representations and delay context effects to relatively late processing. Other, parallel-interactive theories are not architecturally restricted (see also Anderson et al., 2011) and foreground a rapid interaction between syntactic and non-syntactic information sources (e.g., MacDonald et al., 1994; Trueswell and Tanenhaus, 1994). In these, the input is analyzed and ranked according to competing probabilistic constraints. However, constraint-based accounts only hold when a linguistic competitor is present; in this situation, they can select between two competing interpretations (i.e., they do not actively build an interpretation). Moreover, in their present form they do not feature non-linguistic social and contextual representations (see also Novick et al., 2008). Note that we are not claiming that these accounts, models or theories could not be adapted to include social context. We have selected one account (see Guerra and Knoeferle, 2014, 2017; Knoeferle et al., 2014 on how the CIA can accommodate context effects in written language comprehension; see Knoeferle and Crocker, 2006, 2007 for context effects in spoken language comprehension) to illustrate how enrichment with socially relevant information might be achieved.

In summary, extant approaches focus on accommodation, alignment and priming as mechanisms (see Giles et al., 1991; Pickering and Garrod, 2004); on the embodied (vs. abstract) nature of mental representations (see e.g., Glenberg and Robertson, 1999; Zwaan, 2003); and on when linguistic and non-linguistic information influence syntactic structuring (Frazier and Fodor, 1979; Trueswell and Tanenhaus, 1994). Situation models specify protagonists and one could imagine extending them to include comprehender characteristics. The interactive alignment framework accommodates dialog and could also be extended with (speaker and) comprehender characteristics. Yet, it does not include explicit representations of the non-linguistic context nor does it model the interactions of such non-linguistic representations with language processing mechanisms, such as semantic interpretation and syntactic structuring, as comprehenders build an interpretation.

There are, however, accounts (Knoeferle and Crocker, 2006, 2007; Altmann and Kamide, 2007; Knoeferle et al., 2014) and computational models (Mayberry et al., 2009; Crocker et al., 2010) that include at least (representations of) actions and events in addition to objects as visual contexts [but neither information of the comprehender (1) and (2)] nor representations based on the socially interpreted context (3). The Coordinated Interplay Account moreover specifies the linking between (visual) context and how an interpretation of language is derived in real time (Knoeferle and Crocker, 2006, 2007; Knoeferle et al., 2014). But it does not yet accommodate fully explicitly how language processing mechanisms interact with characteristics of the comprehender [see (1) and (2)] and the speaker [captured in (3)].

## Empirical Evidence

By contrast with the state of the art of *modeling* real-time situated language comprehension (theoretically or computationally), much empirical research on (real-time) adult language processing has begun to investigate the effect of (1)–(3) on language comprehension. We next review a selection of offline (picture-choice) and real-time (response times, eye-tracking, and EEG) results that speak to the effects of comprehender characteristics and of the socially interpreted context (including the speaker) on language comprehension.

### Picture-Choice and Response Time Studies

Ethnicity and visual appearance (hinting at socioeconomic status) but also a speaker’s speech style as interpreted by the comprehender (3) can influence a comprehender’s judgment and reaction to linguistic input. Staum Casasanto (2008, 2010) for example showed that a speaker’s ethnicity and speaking style can affect a comprehender’s resolution of referential ambiguity (Staum Casasanto, 2008, 2010). In her first experiment (Staum Casasanto, 2008, 2010), participants read a sentence with either a deleted consonant (*The mis’ predicted by the weatherman surprised me*) or the same sentence without a deletion (*mist* instead of *mis’*). Participants saw a photo of a White person and a photo of a Black person above the written sentences, and circled the picture of the person they imagined uttering the sentence. For sentences with (vs. without) a consonant cluster reduction, participants were significantly more likely to circle the photo of the Black person. Thus, they appear to have implicit knowledge that the ethnicity of the speaker correlates with *t/d* deletion. Reaction time measures in her second experiment revealed that participants reacted faster to the end of a spoken temporally ambiguous sentence (*The mass/mas[t] probably lasted…*) that supported the *mast* interpretation (*…through the storm*) when they saw a Black (than White) face and faster to a sentence ending that supported the *mass* interpretation (*…an hour on Sunday*) of the sentence when they saw a White (than Black) face. In sum, language use can modulate people’s assumption about speaker ethnicity and seeing a speaker of a specific ethnic origin can in turn modulate how quickly a comprehender understands related language.

In a further set of reaction time and picture choice studies, Squires (2013) assessed the links between subject-verb agreement and the inferred social status of the speaker (3) of an utterance on the listeners’ comprehension. Using two non-standard subject-verb agreement variables (NP_[singular]_ + *don’t; there’s* + NP_[plural]_, attributed to working class speakers and an informal register), in her first experiment, Squires primed participants with a low (or high) status picture of a speaker. This picture was presented together with a non-standard (or standard) subject-verb agreement sentence, i.e., low status pictures were combined with non-standard agreement sentences and high-status pictures were combined with standard subject-verb agreement sentences. Squires measured response times and picture choice in a target trial in which participants listened to a sentence in which the grammatical subject of the sentence was covered by white noise (e.g., *In the yard, the [white noise] don’t sit on the feeder*) while inspecting a picture of a low (or high) status speaker. Below the speaker picture, participants saw two photos, one matching the non-standard agreement interpretation of the sentence (e.g., one bird) and one matching the standard agreement interpretation (e.g., many birds). Participants selected the picture they thought matches the sentence best. In experiment 1, participants were more likely to choose a non-standard agreement picture (i.e., depicting one bird) in the presence of a low (vs. high) status speaker picture. By contrast, speaker status did not affect their selection times. Experiment 2 asked participants to choose the speaker depiction (high- vs. low-status) that best matched the sentence (standard vs. non-standard subject-verb agreement). In experiment 2, participants were more likely and also faster to choose the low-status speaker picture when they were primed with a different low-status speaker and non-standard agreement prime (compared with a different high-status speaker and standard agreement prime). Additionally, the results were weaker for *don’t* than for *there’s* trials: Squires suggested that participants may have had weaker sociolinguistic knowledge for *don’t* than *there’s* trials.

Use of the non-standard apical ING-form can also rapidly affect comprehender’s language interpretation and their evaluation of the suitability of an interviewee as a news broadcasting agent [(3), Labov et al., 2011]. Participants from different regions in the United States listened to 10 sentences in a news reading of the same speaker and simultaneously moved a slider on a continuous scale ranging from “try some other line of work” to “perfectly professional” evaluating the speaker’s professionalism throughout the reading. The results indicate a correlation between participants’ negative evaluations and the occurrences of the non-standard apical ING-form. Each time participants heard a non-standard ING-form, suitability ratings dropped toward “try some other line of work.” Labov et al.’s (2011) work shows that adults are highly sensitive in evaluating a speaker’s use of language (the informal ING-form). This effect seems to be more pronounced in women than in men (women reacted more negatively to deviations from the standard use of the ING-form than men).

The results by Staum Casasanto rely on offline (picture choice) and online (response time) measures. Squires (2013) also relies on reaction time and picture choice measures. Labov et al.’s (2011) study – by virtue of measuring participants’ ratings – further suggests a close coupling between the perception/social interpretation of context and language interpretation. Although these experiments have uncovered – at least in parts – rapid speaker effects (ethnicity, visual appearance hinting at socioeconomic status, and speaking style) on a comprehender’s language processing, the measures employed did by and large not track cognitive activity continuously over the time course of sentence presentation. Hence, these studies do not provide us with insights regarding the close temporal coordination between language comprehension and the processing of information from the socially interpreted context [(3), including their modulation by (1) and (2)].

### Continuous Measures: Eye Tracking and EEG

Measures which track cognitive activity over time complement the insights from offline measures. From continuous measures, we can learn about the extent to which a comprehender’s characteristics, but crucially also his interpretation of the context [(3), including for example referents, actions, events, gestures, emotional facial expression and speaker-related information] can affect real-time language comprehension.

For instance, a comprehender’s level of education (e.g., literacy) influences his real-time comprehension and visual attention (Mishra et al., 2012). Two groups of individuals (at high vs. low levels of literacy) listened to spoken Hindi sentences (e.g., *You will now see a tall door*) containing a target word, e.g., *door*, and a restrictive, gender-marked and associated adjective (*tall*, restricting attention to the door since it was the only object out of four available ones that matched in height and gender). Upon hearing *tall* in the sentence, participants with a high level of literacy visually anticipated the door (but not the other objects). The same visual anticipation did not emerge in the individuals with a low level of literacy who shifted their gaze to the door only as it was named. Thus, comprehender characteristics (literacy) modulated the time course of eye fixations to depicted objects during utterance interpretation.

Another experiential characteristic of the comprehender (2) – his mood – can also rapidly affect his language processing. Mood, unlike emotions (e.g., happiness, as elicited by socially relevant events) is usually *not* strongly associated with objects or events and is less prone to rapid fluctuations (cf., Forgas, 1995; Scherer, 2005). Yet, the mood we are in at a given time seems to influence our style of thinking. Being in a good mood seems to result in a more global and anticipatory style of thinking, while being in a bad mood seems to enhance participants’ attention to detail and a focused style of thinking (cf., Zadra and Clore, 2011; Shiota and Kalat, 2012 for reviews). In one study, participants’ mood was induced to be positive or negative via short movies (Van Berkum et al., 2013). Afterward participants read short text passages which (dis)confirmed verb-based expectation about the subject of the subordinate clause. Implicit-causality verbs like *praise* cued information about the second-mentioned character (e.g., *Linda praised David because he had done well*) and verbs like *apologize* cued information about the first-mentioned character (e.g., *Linda apologized to David because she made a mistake*, Garvey and Caramazza, 1974; Van Berkum et al., 2013). Van Berkum et al. (2013) predicted that only when in a good mood, participants would anticipate the next character based on implicit causality (and thus react to a mismatch in verb-based expectation and subordinate clause subject). Indeed, when they were in a good (vs. a bad) mood, participants exhibited a widely distributed increased positivity between 400 and 700 ms to bias-inconsistent (in contrast to bias-consistent) personal pronouns. This demonstrated that induced mood can rapidly and incrementally affect expectations about the grammatical subject of a sentence (see Van Berkum et al., 2007).

Additionally, mean amplitude N400 differences emerged when sentence content conflicted with the comprehenders’ own moral and ethical views (2). Van Berkum et al. (2009) measured participants’ brain waves while they read statements that either were or were not in line with their (pre-tested) ethical views (e.g., *I think euthanasia is an acceptable/unacceptable…*). Value-inconsistent words (e.g., *acceptable/unacceptable*) elicited a small but reliable N400 increase in contrast to words matching comprehenders’ moral and ethical views. Relatedly, Van Berkum et al. (2008) postulated that language interpretation cannot be studied separately from social aspects. Using ERPs, they investigated at what point in time and how comprehenders integrate a speaker’s voice characteristics (3) with their socially- based experience (2). Sentence content matched (or didn’t match) the speaker’s voice (e.g., mismatches: *Every evening I drink some wine before I go to sleep* spoken in a young child’s voice, matches: *Every evening I drink some wine before I go to sleep* spoken in an adult’s voice). The inconsistent sentences contained a word that violated the “probabilistic inferences about the speaker’s sex, age, and socio-economic status, as inferred from the speaker’s voice” (Van Berkum et al., 2008, p. 581). Mean amplitude N400s to the onset of the critical word (*wine*) were larger to mismatching than matching trials, an effect which has also been observed (with highly similar centro-parietal topography) for semantic interpretation in strictly linguistic contexts. This suggests that information from a socially interpreted context is on a par with lexical semantic information – a further argument for including socially interpreted context in accounts of language processing.

Furthermore, age-dependent (1) emotional preferences can affect visual attention and incremental language comprehension in real time. In visual inspection behavior, younger adults showed a preference to inspect negative pictures and faces more than positive ones (i.e., the so-called “negativity bias”); older adults showed a preference toward positive pictures and faces (i.e., the so-called “positivity bias,” see e.g., Socioemotional Selectivity Theory: Carstensen et al., 2003; Isaacowitz et al., 2006). In a visual world eye-tracking study, Carminati and Knoeferle (2013, 2016) and Münster et al. (2014) asked whether this bias generalizes to language comprehension: Younger and older adults inspected a positive or negative emotional prime face of a speaker (3) followed by a negatively and a positively valenced event photograph (presented side by side) and a positively or negatively congruent sentence describing one of the two events (Carminati and Knoeferle, 2013, 2016; Münster et al., 2014). Older and younger participants fixated the corresponding emotionally congruent event photograph more when language and the speaker’s prime face matched (than mismatched) their emotional bias.

Clearly then, characteristics and experience of the comprehender, i.e., (1) and (2) but crucially also a comprehender’s socially based interpretation of the context (3) have an impact on real-time language processing, and yet, the ‘social’ is largely absent from existing accounts of real-time language comprehension. Next, we introduce the social Coordinated Interplay Account (sCIA), an extension of the Coordinated Interplay Account (CIA) toward socially situated comprehension. We outline how we have adapted the CIA to accommodate the effects of (1) – (3) on incremental language comprehension.

## The Social Coordinated Interplay Account

In order to accommodate the findings reviewed above, we can enrich existing accounts of the interaction between contextual cues and real-time language processing with social information. One suitable account is the Coordinated Interplay Account (CIA) by Knoeferle and Crocker (2006, 2007), since it accommodates the interplay of language comprehension with (visual) attention, and already includes non-linguistic context (e.g., objects and events). The 2007 version explicitly included comprehender’s working memory (to accommodate representations of recent visual contexts and their decay). Ensuing research (2014 version, see Knoeferle et al., 2014) accommodated the results from an event-related brain potential study including variation by working memory capacity (high vs. low), overt verification responses, and ERP congruence effects in a picture-sentence verification task. However, these CIA versions explicitly accommodated neither effects of socially interpreted context nor of comprehender characteristics beyond working memory.

**FIGURE 1:**
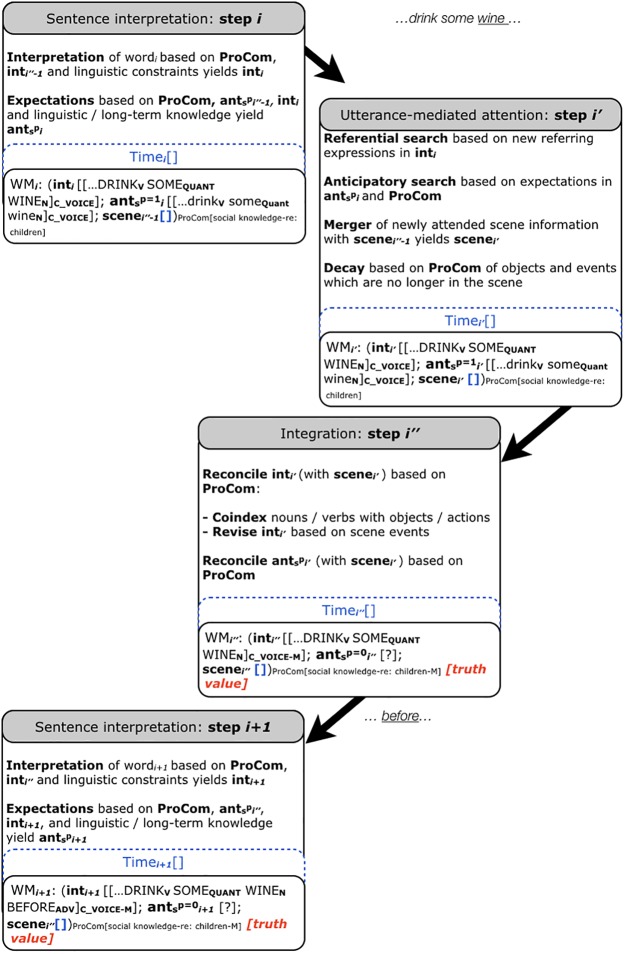
The social Coordinated Interplay Account. The sCIA presents a possible extension of the CIA (Knoeferle and Crocker, 2007; Knoeferle et al., 2014). ProCom can influence the interpretation (int) and (social-) context expectations (ant_s_) that can influence sentence processing in real time. We exemplify the sCIA using the results by Van Berkum et al. (2008). Note that we are illustrating only the processing of the critical word *wine* in *Every evening I drink some…* and assume that the previous input has already been processed. In rendering the mental representation, we present the interpretation starting from the word *drink*. Note also that the sCIA only allows one word at a time to enter the processing cycle. _C_V OICE_ indicates that the comprehender hears a child’s voice. Timing of stimulus presentation has not been manipulated in Van Berkum et al. (2008), hence the Time slot in the account is not filled. Since no visual scene is present in Van Berkum et al. (2008), the scene representation in this example is empty.

The CIA consists of three temporally dependent processing steps that can overlap and occur in parallel. Step i (sentence interpretation) deals with the incremental interpretation of the incoming linguistic input. Here, the input, i.e., a word (word_i_), is interpreted on the basis of the existing interpretation and linguistic constraints, yielding int_i_. Moreover, int_i_, linguistic and long-term knowledge as well as previously established expectations can create expectations (ant_i_). The working memory (WM) comprises both scene- and utterance-based representations for each processing step of word_i_. Step i′ (utterance-mediated attention) accommodates that the interpretation guides attention in WM_i_′ and in the co-present scene. A merger additionally combines information from the newly attended scene with the scene in WM_i′_ (scene_i′′-1_), whereby ‘-1’ indicates the previous processing cycle. The merger yields scene i′. Next (step i″, scene integration), the interpretation int_i′_ and the expectation ant_i′_ are reconciled with the scene_i′_. The outcome of this reconciliation is tracked (match vs. mismatch) via indices to ant and int, and a truth value tracks the veracity of manual responses. Regarding int_i′_, reconciliation involves coindexing representations of (minimally) nouns/verbs with those of depicted objects/actions (see Guerra and Knoeferle, 2014, 2017 on non-referential co-indexing). Additionally, revision of the sentence interpretation can be informed by the scene. When the comprehender encounters the next word, the interpretation is updated, taking the previous expectations and interpretation into account (step i+1, Knoeferle and Crocker, 2007). Note that the CIA (and thus also the sCIA) does not require a visual scene to be present to function. In **Figure 1** this is represented by the empty scene [].

**FIGURE 2:**
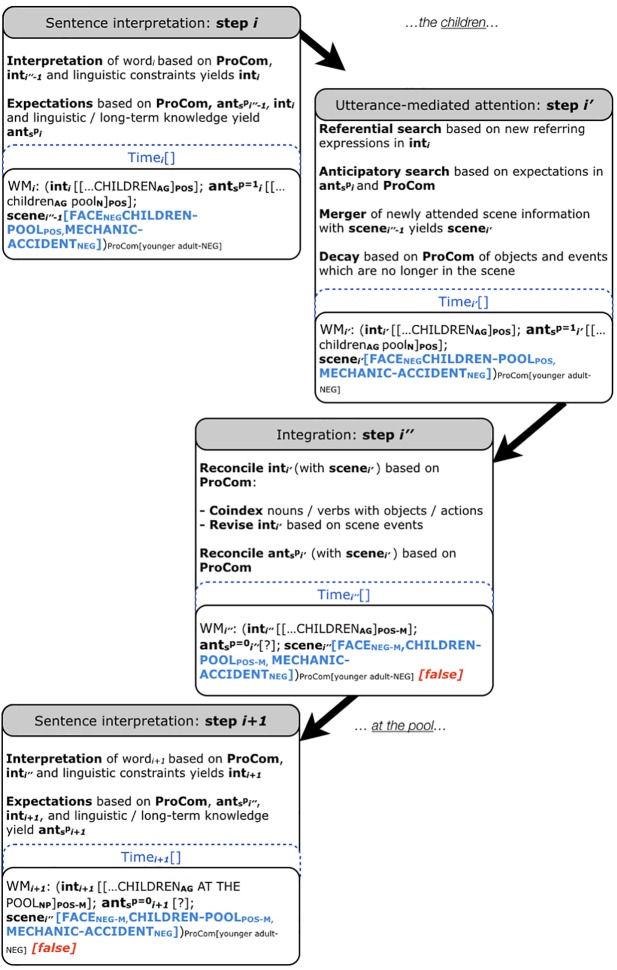
The social Coordinated Interplay Account. We exemplify the sCIA using the results for the mismatching speaker face – sentence valence condition for younger adults by Carminati and Knoeferle (2013). The original sentences were in German, we are using the literal English translation here. Note that we are illustrating only the processing of the first word which gives away the mismatch in emotional valence between the speaker’s facial expression (negative) and the picture that the sentence is about (positive), i.e., *children* in *I think that the children at the pool…*. We assume that the previous input has already been processed. In this study, a visual scene is present and hence ‘scene’ is filled. Moreover, an overt response is required, hence the ‘[truth value]’ is set to ‘[*false*]’ for the mismatch detection.

The social Coordinated Interplay Account (sCIA, **Figures 1**, **2**) adds three extensions to the CIA (Knoeferle and Crocker, 2006, 2007; Knoeferle et al., 2014):

Extension 1: ‘ProCom’ (see **Figures 1**, **2**). ProCom is a variable that contains the properties of the comprehender, viz. the biological (1) and experiential (2) characteristics introduced above. Note that previously, the only property of the comprehender included in the account was working memory (first introduced in the 2007 version and specified as ‘high’ vs. ‘low’ in the 2014 version). The present extension toward ProCom has the effect that working memory is one of several properties of the comprehender captured by ProCom (see **Figures 1**, **2**). The comprehender characteristics can affect language processing in context (3). The CIA featured reconciliation of int and ant with the scene in step i″; the sCIA adds reconciliation possibilities of int and ant with ProCom in step i″.

Extension 2: Speaker characteristics, marked in **Figures 1**, **2** as indices and representations in ‘scene’.

Extension 3: The sCIA (see **Figures 1**, **2**) extends ‘ant’ of the CIA, yielding ‘ant_s_’. Recall that ‘ant’ comprises the expectations of the comprehender in the CIA. The sCIA enriches it with expectations derived from the socially interpreted context [(3) e.g., from referents, actions, events, gestures, emotional facial expression and speaker voice], yielding ant_s_. Probabilities ranging from 0 to 1 capture the graded nature of the expectations in ant_s_. In **Figures 1**, **2** we are setting these probabilities to 1 and 0 for illustration purposes. They capture the activation ^p^ of expectations ant_s_, whereby ^p^ can be modulated by ProCom and the socially interpreted context during language processing.

### The sCIA – Example

We exemplify how the sCIA functions by accommodating the results from four experiments. Recall that Mishra et al. (2012), using eye tracking, tested individuals (with a high vs. low level of literacy) on their visual anticipation and expectations regarding the interpretation. Upon hearing *tall* in Hindi sentences (e.g., *You will now see a tall door*.), individuals with a high level of literacy anticipated the door (vs. three distractor objects not compatible with *tall*). By contrast, individuals with a low level of literacy shifted their gaze to the door only later, as it was named, even when *tall* had restricted the domain of reference to the door. Mishra et al. (2012) suggest that reading and writing skills might fine-tune anticipatory mechanisms and hence modulate the prediction skills of individuals with a high- and low level of literacy. In the sCIA, literacy – as a property of the comprehender (2) is part of ProCom (here: high vs. low literacy although more graded notions would be possible) and can modulate the probabilistic ^p^ of ant_s_. This effect could be captured in step i′ when the incoming word *tall* in *You will now see a tall door* mediates attention to the objects in the scene. For the individuals with a low level of literacy, ProCom sets ^p^ of ant_s_ to a lowish value (closer to zero than to one), effectively modulating the generation of expectations. For the individuals with a high level of literacy, by contrast, ProCom would set ^p^ to a high value (closer to one than to zero, depending on educational background), leading to clear expectations of the mention of upcoming objects upon encountering the restrictive adjective. For individuals with a high level of literacy, one could imagine that the connections between the mental representations of ‘tall’ and ‘door’ (supported by the visual context) are stronger than for individuals with a low level of literacy (since individuals with a high level of literacy could activate both the phonological form and the written form, perhaps boosting semantic and syntactic representations and eliciting earlier anticipatory eye movements to the door). Alternatively, what differs by literacy is not the mental representations of ‘tall’ and its connection to ‘door’ (and its referent) but how rapidly – once the representation of ‘tall’ has been accessed – comprehenders draw inferences regarding soon-to-be-mentioned objects.

Recall a further example in which participants’ moral and ethical views (i.e., as elicited by sentences about ethical topics such as *euthanasia is an un/acceptable practice*) influenced language comprehension in real time (Van Berkum et al., 2009). Comprehenders’ sentence reading was only affected by the moral content once they had encountered the critical word (*un/acceptable*). In the sCIA, ProCom captures the comprehender’s experiential knowledge (2). This experience-based knowledge is activated by the first word (*euthanasia*) in step i of the sCIA. The comprehender expects the subsequent input to be in line with his social views (i.e., if he is against euthanasia, he may expect ‘unacceptable’ more than ‘acceptable’) and generate corresponding expectations captured by ant_s_. Depending on the ensuing input, these expectations increase or decrease as the utterance unfolds. In the present example, in step i″, the interpretation and expectations are each reconciled based on ProCom. To the extent that the expectations become more specific, the value ^p^ for ant_s_ would increase. Upon reading *(un)acceptable*, the comprehender’s expectations ant_s_^p^ (informed by ProCom) mismatch (vs. match) during reconciliation with the representation of the sentence (step i″). This mismatch between the comprehenders’ expectations informed by ProCom and the semantic meaning of the sentence established by the target word elicits in the account an N400 effect similar to a semantic N400 effect. Underlying the probabilistic expectations could be mental representations that link the representation of ‘euthanasia’ to the lexical representation of ‘acceptable’ (or ‘unacceptable’). If so, the comprehender would potentially derive lexically specific expectations. Alternatively, or in addition, the mental representation of ‘euthanasia’ could connect to valenced representations of events that the comprehender has directly or indirectly experienced in life and that have influenced the comprehender’s value system regarding euthanasia. In the latter case, the comprehender might anticipate the valence of the associated event (as opposed to a specific word; see Van Berkum et al.’s interpretation of the results; Van Berkum et al., 2009). One could also imagine a linking in which the lexical representation is activated first, eliciting the activation of further event representations. Or, conversely, event-representation much like ‘gist’ might be activated top-down, with the activation of specific lexical representations following suit.

Recall as a third example that Van Berkum et al. (2008) manipulated speaker voice and word congruence (e.g., *Every evening I drink some wine before I go to sleep* spoken by a child vs. by an adult) in a sentence comprehension ERP study. Mean amplitude N400s increased when the listener’s expectations about sentence content (motivated by a child’s vs. an adult’s voice) mismatched (vs. matched) a critical word (e.g., *wine* would jar for a sentence spoken by a child). ProCom captures the comprehender’s experience-based social knowledge (2) and can modulate ant_s_ (see **Figure 1**). Ant_s_ comprises the listener’s social expectations elicited by the speaker’s voice (3) regarding sentence content (i.e., about what a child drinks before going to bed when the speaker’s voice is that of a child). The value of ^p^ for ant_s_ would increase as expectations of the sentence content become more specific (i.e., as listeners hear *drink some*, at which point they likely expect a non-alcoholic drink). Upon encountering the word *wine*, and following the attention-mediated step, int_i′_ and ant_s_^p^_i′_ are each reconciled based on ProCom in step i″. Resulting from this reconciliation is incongruence with the experience-based knowledge of the comprehender [as marked by M (mismatch) in **Figure 1**], leading to processing differences compared to matches (i.e., had the same sentence been spoken by an adult). Note that the weight ^p^ assigned to ant_s_ can vary in strength depending on, for example, a listener’s previous experience with relevant social situations (2). This means that we would expect different results, i.e., a reduced or even absent N400 effect depending on cultural differences (e.g., if it was not uncommon in a certain culture for children to drink wine to help them sleep or if the comprehender were a child with limited experience about the socially acceptable wine drinking age). With regard to possible underlying mental representations, the voice of the speaker might evoke representations of speaker identity (in the case of this example a child). These representations could then be enriched through the semantic content of the sentence (here: a routine of drinking something every evening), leading the comprehender to construct a mental representation of a situation in which a child is involved in a daily evening routine of drinking something. This mental representation could activate associations to likely candidates of this particular drinking action given the particular situation. The comprehender might then anticipate *milk* or another non-alcoholic drink as the most likely candidate. However, when the constructed mental representation of a child who drinks a glass of milk every evening is violated by the inconsistent word *wine*, the reconciliation of the expectations (that were set up for the next upcoming word) with the newly encountered information (*wine*) is costly and leads to the observed N400 increase (at step i″ in the sCIA).

While we have illustrated how ProCom moderates linguistically mediated knowledge in the sCIA (**Figure 1**), the sCIA is fundamentally an extension of the CIA which was developed to accommodate effects of the non-linguistic *visual* context. Accordingly, the sCIA can accommodate (expectation-mediated) effects of the socially interpreted visual context: **Figure 2** illustrates an example for a mismatch between a facial speaker expression (negative, _‘NEG’_) and sentence valence (positive, _‘POS’_). The subscripts _‘NEG-M’_ and _‘POS-M’_ mark the mismatching representations in **Figure 2**. Recall that Carminati and Knoeferle (2013) reported that effects of the speaker’s facial mimics on language comprehension varied by comprehender age. The task in their study was to “look, listen and understand the sentence, and decide whether the valence of a speaker’s face matched the valence of the sentence” (p. 6). For younger adults (18–31 years of age), a match between a negative (vs. positive) facial expression and negative sentence valence boosted looks to a negative event photograph as soon as valenced utterance information became available. By contrast, a match between a positive (vs. negative) facial expression and positive sentence valence did not boost looks to a positive event photograph. The older adults experienced a boost in attention for positive–positive but not negative–negative face-sentence matches. In the sCIA, ProCom captures the age and its associated emotional bias (marked by _‘ProCom[younger adults-NEG]’_). ‘Scene’ captures the scene representations (as was the case in the CIA). It is possible that (representations of) the scene received differential attention by age, leading to a decay of negative (positive) facial expressions in older (younger) adults, and thus a reduced attentional boost. Alternatively, valence-marked sentence representations received differential attention by age, guiding attention to co-present event photographs and their corresponding representations (differentially so by age). These modulations of visual attention could be captured by the effects of age and age-related emotional biases on ant_s_^p^.

In conclusion, the sCIA presents the first step toward a dynamic, word-by-word sentence comprehension account that can accommodate how comprehender characteristics and a (comprehender’s interpretation of a) social context modulate real-time language comprehension. Future extensions could accommodate context effects also below the word level, subtle variations in situation-specific language style, as well as additional experimental results at the word, sentence, and discourse level for mono- and bilingual comprehenders.

## Discussion and Future Directions

In the present paper, we have argued for the integration of socially interpreted context (including speaker information) and comprehender characteristics into accounts of the interaction between language processing and (visual) attention. Extant processing accounts are underspecified regarding the integration of social (speaker) information and comprehender characteristics. We presented an extension of the CIA (Knoeferle et al., 2014) and demonstrated how the extended social Coordinated Interplay Account accommodates recent findings (Van Berkum et al., 2008, 2009; Mishra et al., 2012; Carminati and Knoeferle, 2013).

Integrating these kinds of social information into the CIA is the first step toward acknowledging the impact of “the social” on language comprehension. The present paper has taken a comprehender-centric viewpoint. We acknowledge that much remains to be done, such as extending accounts of real-time language *production* with representations of social information. The overarching goal would then be to bridge the comprehension and production accounts and integrate them into a higher-level situation framework, such as the one by Zwaan (2003) or by Pickering and Garrod (2009, 2013). In his framework, Zwaan (2003) suggests an interaction between abstract and grounded symbols. The interaction of these symbols varies with the situation. Words and their referents in the real world are encountered in spatio-temporal settings. These settings feature objects, agents and events, which in turn co-occur with referents. A situation framework (e.g., Zwaan, 2003) could accommodate a real-time processing account, such as the sCIA. A computational implementation of the latter (e.g., extending Mayberry et al., 2009; Crocker et al., 2010) would permit more precise simulation of the results and predictions for future empirical investigations.

## Author Contributions

KM reviewed the literature. KM conceptualized a first version of the sCIA (Münster, 2016) and provided a first draft of the manuscript. PK and KM discussed and substantially revised the sCIA and the manuscript.

## Conflict of Interest Statement

The authors declare that the research was conducted in the absence of any commercial or financial relationships that could be construed as a potential conflict of interest.

## Footnotes

1 We acknowledge the role of speakers as active participants in communication. However, in this paper we focus on real-time language comprehension, leaving language production aside.
